# Consensus on the Screening, Staging, Treatment, and Surveillance of Localized, Recurrent, and Metastatic Prostate Cancer: The First Global Prostate Cancer Consensus Conference for Developing Countries

**DOI:** 10.1200/GO.20.00518

**Published:** 2021-04-15

**Authors:** Fernando Cotait Maluf, Silke Gillessen

**Affiliations:** ^1^ Hospital Israelita Albert Einstein, São Paulo, Brazil; ^2^ Beneficência Portuguesa de São Paulo, Brazil; ^3^ Latin American Oncology Group (LACOG), Porto Alegre - RS, Brazil; ^4^ Oncology Institute of Southern Switzerland (IOSI), Bellinzona and Università della Svizzera italiana, Lugano, Switzerland; ^5^ Manchester Cancer Research Centre, Division of Cancer Sciences, University of Manchester, United Kingdom

## INTRODUCTION

Prostate cancer (PCa) incidence corresponds to 1,276,106 new patients yearly according to the Globocan 2018 and 358,989 of deaths with 70% of them occurring in more developed regions.^1^ The highest incidence rates have been reported in North America, Oceania, and Northern and Western Europe.^2^ Fortunately, the PCa mortality has been declining in most Western countries and in some European countries such as Finland, Sweden, Portugal, Israel, Italy, the Netherlands, Norway, and France probably because of screening, early detection, and advances in local and systemic treatments.^3^

On the other hand, many developing countries in Latin America such as Colombia, Costa Rico, and Ecuador have experienced increasing PCa trends from 1993 to 2002, and countries such as Brazil, Colombia, Cuba, and Ecuador have reported an increase in mortality rates.^4-6^ Between 2005 and 2009, Cuba, Uruguay, and Venezuela had the highest PCa mortality rates in the Americas of more than 18 per 100,000 overall. Chile, Argentina, Costa Rica, Puerto Rico, Colombia, and Brazil had overall rates between 13 and 17 per 100,000 during the same period.^7^ Similarly, Asian countries such as Thailand and Korea have reported an increase in mortality rates from 50% to 260%, respectively.^8^ In the same direction, Center et al^4^ have reported a rapid increase in mortality in most Central and Easter European countries.

Although the cancer-specific mortality has been reported to be the lowest in Asia and Northern Africa, Chu et al^9^ have reported that advanced-stage rates in East Africa have been much higher than those of African Americans in recent years. As an example, Osegbe^10^ reported that 64% of patients with new PCa in a Nigerian hospital died within 2 years of diagnosis. It has been estimated that 57,048 deaths associated with PCa will occur in Africa by 2030. This represents a 104% increase in the number of PCa deaths in Africa over the next 20 years.^11^

A total of 28,000 deaths of men of all ethnicities in Africa because of PCa as reported were more than four times the 6,500 that occurred among Caribbean men of all ethnicities and exceeded deaths from any other cancer in Africa.^12^

There are multiple reasons aside from variation in tumor biology for the increased mortality rates in developing countries, which include lack of a screening program, diagnosis at an advanced stage, limited access to local and systemic treatments, most affected being urban poor or from rural and remote populations having poor or no healthcare coverage, lack of affordable transportation to health centers, limited number of critical therapeutical tools such as radiation therapy, missing specifically trained healthcare staff including physicians with not a full training in oncology, and lack of information and guidelines. Many guidelines have addressed the gold standard in the diagnosis and management of PCa such as the National Comprehensive Cancer Network guidelines, but in general they do not provide specific, detailed, and adapted information and guidance for low-resources areas.^13^

## METHODOLOGY

The first Global Prostate Cancer Consensus Conference for Developing Countries (PCCCDC) was organized around state-of-the-art lectures and presentations and discussed evidence relevant to 12 key topics and subtopics related to the management of PCa in general and in limited-resource regions (screening, diagnosis, staging tools, treatment, and follow-up for various stages of cancer). Four polling sessions were scheduled during the 2-day conference for panelists to respond to questions regarding these topics. Only physicians who participated in all four sessions are included in the final consensus results. Several of the questions were based on the questions from the Advanced Prostate Cancer Consensus Conference (APCCC) 2017, the conference that stimulated and inspired the PCCCDC.^14^

The full panel for this consensus paper consisted of 99 multidisciplinary cancer physicians including urologists, medical oncologists, radiation oncologists, radiologists, and pathologists from developing countries in Latin America, Africa, Middle East, Asia, and Eastern Europe. The panel members were selected on the basis of recent work and their willingness to be present at the first PCCCDC. The draft of recommendations and clinical questions were selected previously by a committee composed of specialists in the fields. The committee of radiologists was led by Douglas Racy, MD; the committee of radiotherapy was led by Robson Ferrigno, MD; the committee of urology was led by Arie Carneiro, MD, Murilo Luz, MD, and Gustavo Guimarães, MD; the committee of oncology was led by Fernando Cotait Maluf, MD, Felipe Moraes, MD, and Andrey Soares, MD.

**FIG 1:**
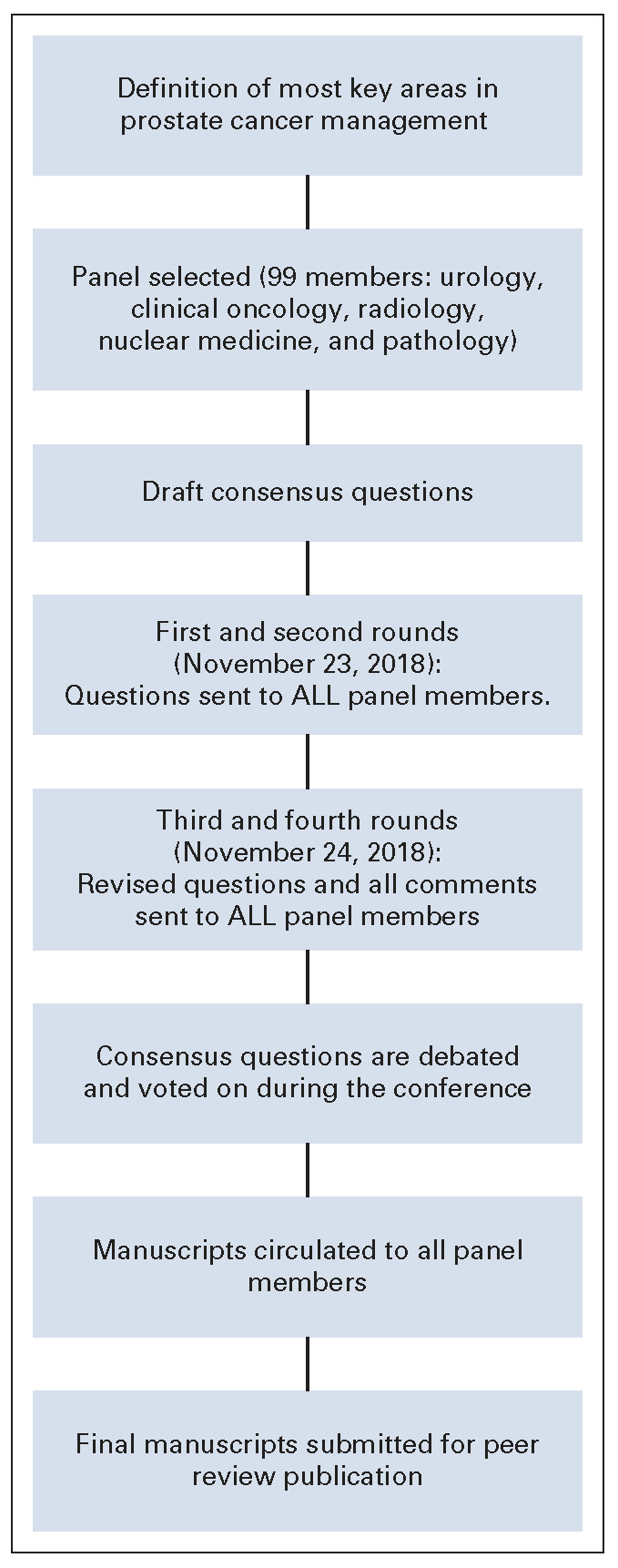
Modified Delphi process for consensus.

The consensus was developed on the basis of a modified Delphi approach (Fig 1). In a modified Delphi process, questions based on the sections were created and in the first round sent to all panel members. Questions and options for answers were later revised and sent a second time to all panel members, including all inputs received shown in an anonymized fashion, so all the panelists could see every comment from their colleagues. The comments from the second round were included in the third version that was then circulated. After the third round, only critical changes were accepted for the fourth and definitive version of the questions. This process was analogous to the one used for the APCCC 2017.^14^ The conference included presentations and debates from participants who reviewed evidence relevant to the above questions. On the last day of the conference, all questions were presented with options for answers in a multiple-choice format. The questions were voted on publicly but anonymously. In some cases, discussions and revoting occurred. Each question had five to seven answer options including two nonanswers (abstain and unqualified to answer). Each question was deemed consensus if 75% or more of the full panel selected a particular answer. The two nonanswers were provided for quality control. Unless stated otherwise, it is assumed that for the specific recommendation (type of surgery, type of radiation therapy, and drug), therapies are approved and available, no treatment contraindications exist, and no clinical trial is currently ongoing. If consensus is not achieved, then the Steering Committee may opt to leave a clinical question unanswered and state, “Consensus could not be achieved.” All the questions can be accessed in the Data Supplement. For the questions that refer to an area of limited resources, the recommendations consider cost-effectiveness and the possible therapies with easier access. In addition, recommendations are for nonfrail patients and for patients with prostate adenocarcinoma (unless stated otherwise). Their answers are annotated and discussed in the following manuscripts that address screening, diagnosis, and staging tools, and treatments for the topics are stated in Table 1.

**TABLE 1:**
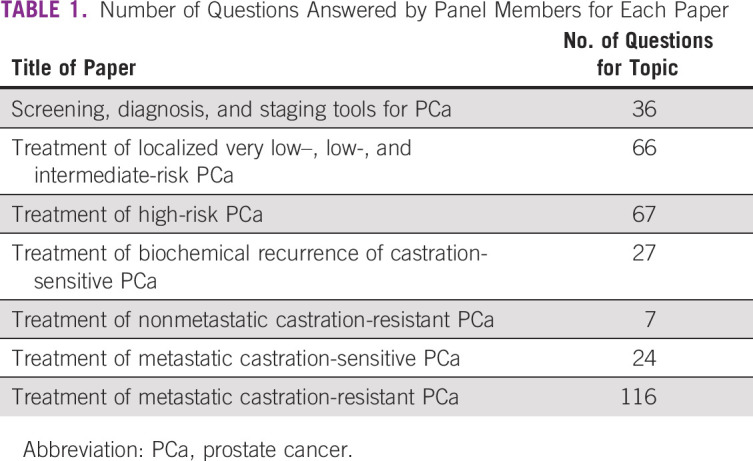
Number of Questions Answered by Panel Members for Each Paper

## PANEL PARTICIPANTS

The panel for this consensus paper consisted of 99 multidisciplinary cancer physicians including urologic surgeons, medical oncologists, radiation oncologists, radiologists, and pathologists from developing countries in Latin America, Africa, Middle East, Asia, and Eastern Europe. The selection of panel members was based on special expertise in PCa, recent work in this field, and their willingness to attend the first PCCCDC. All the participants and medical specialties are included in the Data Supplement.

## QUESTIONNAIRE

The questionnaire was developed by a panel of seven experts to provide relevant real-world physician recommendations for nonfrail patients (as defined by the Eastern Cooperative Oncology Group performance status 0-2) and for patients with prostate adenocarcinoma (unless otherwise stated). A total of 321 questions were constructed to investigate (1) screening, (2) diagnosis, (3) staging tools, (4) treatment, and (5) follow-up of PCa and the impact of limited resources on those treatment recommendations by panelists. For some topic, the questions were based on the APCCC 2017.^14^ Following each question, there were five to seven answers options including the option to abstain and/or to identify that they felt unqualified to answer. These options allowed for physicians to opt out of questions that they may not contend with in their specific specialty. Questions were designed to include recommendations of therapies or treatments that are approved and available, and no treatment contraindications exist, unless otherwise stated in the question. An answer was deemed to have reached consensus if 75% or more of the full panel selected a particular answer. For the questions referring to an area of limited resources, the clinicians were instructed to consider both cost-effectiveness and access.

## ARTICLES

The series includes seven articles compiling all the questions applied on the PDCCCD. The main objective of each article will be discussing the question raised and evaluating the voting panel and literature discussion related to the questions. The first article includes questions of the consensus on screening, diagnosis, and staging tools in PCa. Benefits on screening and early detection of PCa and limitations of screening diagnostic tools in countries of limited resources will be addressed. The second and third articles will include the consensus statement on treatment of localized PCa of low, intermediate, and high risk. In these articles, interventions for localized disease including active surveillance will be discussed, considering life expectancy and disease risk. The fourth article considers biochemical recurrence in castration-sensitive PCa. Challenges in the treatment of this disease rely on preventing or delaying the onset of metastatic disease; morbidity and mortality will be discussed in the article. The fifth manuscript will include questions on nonmetastatic castration resistance PCa, importance of PSA doubling time, and risk assessment. The sixth article will include questions about metastatic castration-sensitive prostate cancer, and the last article will include issues of metastatic castration-resistant prostate cancer. Discussions about staging, sequencing strategies, and limitations of access in areas of limited resources will be dissected.

## OBJECTIVE OF THE SERIES

To provide valuable information for physicians and healthcare professionals who work in areas with limited resources, we sought to provide a global consensus including screening, diagnosis, staging tools, treatment, and follow-up for various stages of PCa for developing countries. The information provided in the consensus posing different scenarios will help to choose, when the gold standard options are not available, the best option possible considering a resource-limited scenario. We hope that the series will help physicians who work treating patients with prostate cancer in areas of limited resources to establish the best possible standard of care for the patients in these countries and help in guiding and educating responsible groups in these countries of the limitations faced by these professionals in the treatment of PCa.
